# A transgenic zebrafish leukemia model driven by *KMT2A::MLLT3 (MLL-AF9)*

**DOI:** 10.1038/s41375-026-02953-y

**Published:** 2026-04-22

**Authors:** Maryam Saberi, Omid Delfi, Christopher J. Hall, Dona W. K. K. Madola, Peter J. Browett, Purvi M. Kakadia, Stefan K. Bohlander

**Affiliations:** 1 Leukaemia & Blood Cancer Research Unit, Department of Molecular Medicine and Pathology, Faculty of Medical and Health Sciences, The University of Auckland, Auckland, New Zealand; 2 Department of Molecular Medicine and Pathology, Faculty of Medical and Health Sciences, University of Auckland, Auckland, New Zealand; 3 New Zealand Blood Service, Epsom, Auckland, New Zealand

**Keywords:** Acute myeloid leukaemia, Cancer models

Acute myeloid leukemia (AML) is a highly aggressive, genetically heterogeneous hematopoietic malignancy driven by the accumulation of mutations in hematopoietic stem cells. A frequent and clinically significant alteration in AML is the t(9;11)(p22;q23) translocation, which generates the oncogenic *KMT2A::MLLT3* (MLL-AF9) fusion [1]. This fusion is found in both de novo and therapy-related acute leukemias, including AML and B-ALL, across pediatric and adult populations, and is associated with poor disease-free and overall survival [2]. Thus, there is an urgent need for novel therapies targeting MLL fusion–driven leukemias, for which robust animal models are essential. Zebrafish (ZF) has emerged as a powerful vertebrate model for hematopoietic disease research as they can be easily genetically manipulated, are optically transparent, develop rapidly, and recapitulate the molecular and cellular features of human hematopoiesis and malignancies [3]. Even though several ZF AML models have been reported, to our knowledge none of the models driven by a single oncogene has been shown to be transplantable [4, 5].

In this study, We generated two independent transgenic zebrafish lines expressing the human *KMT2A::MLLT3* (MA9) fusion oncogene in hematopoietic stem cells under the control of the murine Runx1 + 23 enhancer/promoter, designated as Tg(R:MA9-EGFP) and Tg(R:MA9-mCherry) (Fig. S1). In these lines, MA9 expression was coupled to either EGFP or mCherry via an IRES element. In the first six days after transgene injection, F0 MA9 transgenic larvae exhibited substantial mortality ( ~ 40%), accompanied by morphological abnormalities when compared with wild-type and control transgenic fish lacking MA9 expression. MA9 expression was confirmed in both larvae and adult fish by RT-PCR (Fig. S2).

From approximately six months of age, MA9 transgenic fish began to display overt disease phenotypes, including abdominal distension, gill bleeding, lethargy, and ocular abnormalities (Fig. 1A). Post-mortem examination of affected fish revealed severe pathology consistent with leukemia, including enlarged and abnormal kidneys, splenomegaly with white surface lesions, and widespread disruption of normal kidney and splenic architecture (Fig. 1B, C). Histological analyses showed increased cellularity, disruption of tissue organization, and extensive infiltration of leukemic cells in peripheral tissues, including skeletal muscle (Fig. 1D, Fig. S3).

**Fig. 1:**
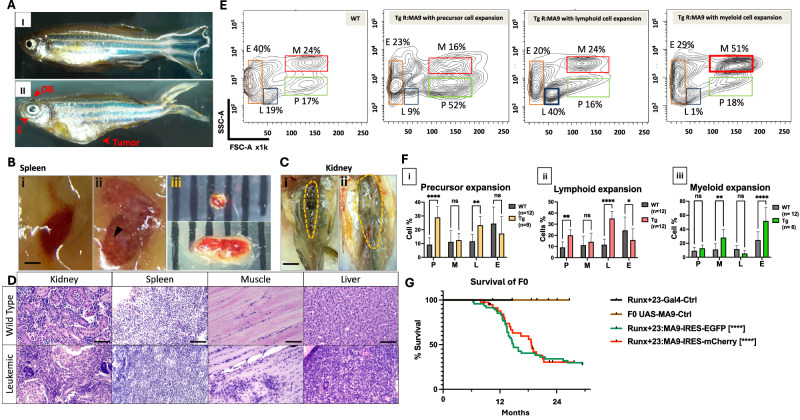
Analysis of the leukemic F0 transgenic Tg(Runx1 + 23:MA9) fish. **A** Morphology comparison in adult fish (I) Ten-months-old wild-type zebrafish under visible light. (II) Ten-month-old leukemic transgenic R:MA9 fish under visible light with expanded olfactory bulb (OB), splayed eye and retro-orbital soft tissues (E) and extended abdomen. Scale bar is 0.5 cm. **B** Sick transgenic fish showed enlarged and pale spleen, often with pronounced white spots. (i) normal spleen in WT fish, scale bar is 0.5 mm (ii) enlarged spleen in R:MA9 F0 fish with white spots (black arrow). (iii) The spleen in leukemic F0 fish (bottom) was pale and three times bigger than in WT fish (Top) (scale in millimeters). **C** Sick transgenic fish showed large, pale and abnormal kidney. (i) Normal kidney in 11 months old wild-type fish. (ii) Pale and enlarged kidney in an 11 months old R:MA9 F0 fish. Yellow dashed line encircles the kidney. Scale bar is 0.5 cm. **D** Histological analysis shows the infiltration of leukemic cells into the kidney, spleen, muscle and liver of leukemic fish (Bottom) compared to WT (Top). The scale bar represents 50 µm. **E** Forward scatter (FSC)/ side scatter (SSC) analysis of the cells from the kidney of wild-type (WT) control and representative F0 leukemic Tg(Runx1 + 23:MA9) fish, showing expansion of different hematopoietic compartments in different leukemic fish, including precursor, lymphoid, or myeloid cells. Gated populations are as follows: myeloid cells (M, red box), erythrocytes (E, orange box), lymphocytes (L, blue box) and hematopoietic precursor cells (P, green box). For each animal, the cell percentages were normalized so that the sum of all gates came to 100%. **F** Comparison of WKM cells profile in WT and F0 Tg(Runx1 + 23:MA9) using flow cytometry. Two-way ANOVA was performed using the Prism 10 Software to find significant relative differences in the hematopoietic cell populations of wild-type (WT) and leukemic (Tg) fish. FSC/SSC analysis was performed on age-matched animals. **, *p* = 0.0073 (precursor expansion); *, *p* = 0.0317; **, *p* = 0.0042 (Lymphoid expansion); **, *p* = 0.0025 (myeloid expansion); ****, *p* = <0.0001. **G** The survival curve of adult F0 Tg(Runx1 + 23:MA9-IRES-EGFP) and Tg(Runx1 + 23:MA9-IRES-mCherry) fish. The Tg(UAS:MA9-IRES-EGFP) and Tg(Runx+23:Gal4) fish were used as controls. Tick marks show the censored fish. Statistical test: two-way ANOVA: ****, p < 0.0001.

Flow cytometric analysis using forward and side scatter of kidney marrow cells from 40 diseased MA9 transgenic fish revealed profound alterations in hematopoietic cell composition relative to wild-type controls. Subsets of fish exhibited significant expansion of precursor cells, expansion of lymphoid populations with concomitant loss of erythroid cells, or the expansion of myeloid compartments (Fig. 1E, F) while others displayed aberrant forward/side scatter profiles indicative of novel cell populations (Fig. S4). Consistent with lymphoid involvement, fluorescent imaging identified green thymi in a subset of Tg(R:MA9-EGFP) fish at leukemia onset, including one case of apparent transmission to offspring (Fig. S5). By two years of age, 77% of F0 MA9 transgenic fish had developed leukemia, as confirmed by histopathology and transplantation assays, whereas nearly all control fish survived beyond this time point (Fig. 1G). Together, these findings demonstrate that hematopoietic expression of human *KMT2A::MLLT3* robustly induces leukemia in zebrafish.

Stable MA9 transgenic zebrafish lines were generated by crossing Tg(Runx1 + 23:MA9-EGFP) or Tg(Runx1 + 23:MA9-mCherry) F0 fish with wild-type fish, and F1 offspring were screened for transgene transmission by fluorescence microscopy and RT-PCR (Fig. S6). Six F0 founders transmitted the MA9 transgene to 15–25% of their offspring, including one line with >50% transmission. MA9 expression was confirmed in adult F1 leukemic fish. Analysis of F1 embryos by whole-mount in situ hybridization revealed reduced expression of erythropoietic markers (scl, gata1) and increased granulocyte markers (mpx, lyz), indicating early hematopoietic disruption (Fig. S7A, B). In-crossing of F1 lines produced F2 transgenic fish. Leukemia developed in 40% of F1 and 28% of F2 animals at median latencies of 23 and 21 months, respectively (Fig. S7C, Fig. S8). This demonstrates efficient germline transmission of MA9 and its leukemogenic potential across generations.

Further, to assess the transplantability of MA9-driven leukemia, kidney marrow (KM) cells from three 12-month-old leukemic Tg(R:MA9-EGFP) F0 fish were transplanted into sublethally irradiated wild-type recipients. After transplantation, 20 of 22 recipients developed leukemia within 17–48 days (Fig. 2A), with MA9 expression confirmed by RT-PCR (Fig. 2B). Post-mortem and histological analyses revealed characteristic leukemic pathology, including enlarged and pale kidneys, splenomegaly (Fig. S9), and extensive leukemic infiltration of the kidney, liver, and muscle (Fig. S10). Flow cytometric and cytomorphological analyses demonstrated expansion of precursor, myeloid, or lymphoid populations, emergence of abnormal cell subsets, and circulating blasts (Fig. 2C, D). Secondary transplantation of KM cells from a primary leukemic recipient resulted in leukemia in all secondary recipients within 27–53 days, confirming robust self-renewal and disease-propagating capacity (Fig. S11,12).

**Fig. 2:**
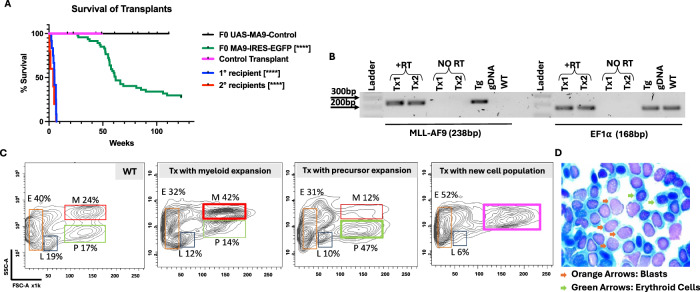
The F0 *MLL-AF9* leukemia is transplantable. **A** Kaplan-Meier survival plot shows a comparison in leukemia latency. Primary (1°) recipients transplanted with WKM cells from F0 donors (blue). The age of the donor at the time of transplantation was 12 months. Secondary (2°) recipients transplanted with WKM cells from 1° donor (red). Blue and red are 1° and 2° KM transplants, with median latencies of 32 and 40 days, respectively, which is a shorter latency compared to the F0 Runx+23:MA9-IRES-EGFP (green), with a median latency of 390 days. Control transplants (pink) and control transgenic F0 UAS-MA9 (grey) didn’t develop leukemia. **B** An agarose gel picture confirming the expression of MA9 in a representative 1° and 2° transplant recipient at the transcript level in the WKM cells. The expression was also confirmed in other samples. Tx1: 1° recipient, Tx2: 2° recipient, + RT: with Reverse transcriptase, WT: cDNA from wild type fish, NC: water as negative control. Tg gDNA serves as the positive control. No bands in No RT reactions confirm the absence of gDNA contamination in the RNA sample. Product in +RT reactions confirms the presence of the MA9 transcript. **C** Flow cytometric analysis on WKM of WT and representative leukemic transplanted fish with myeloid cell expansion, precursor cell expansion, or with a new emerging population (pink). Gated populations are as follows: M: myeloid cells (red), E: erythrocytes (orange), L: lymphocytes (blue) and P: hematopoietic precursor cells (green). **D** Wright-Giemsa stained peripheral blood smear of a 2° leukemic transplant showing increased blasts.

As the expression of the GFP protein in the R:MA9-EGFP transgenic fish was not very strong and often absent in the leukemia cells, R:MA9-EGFP fish were crossed with Ubi:mCherry fish to generate R:MA9-GFP/Ubi:mCherry animals, in which all hematopoietic cells were fluorescently labeled. KM cells from a leukemic R:MA9-GFP/Ubi:mCherry fish were transplanted into 20 irradiated recipients, with engraftment evident within 14 days. Nineteen of 20 recipients developed leukemia within 69 days, displaying diverse hematopoietic phenotypes, including precursor, myeloid, or lymphoid expansion, or the emergence of new cell populations. Secondary transplantation of KM cells from a leukemic primary recipient induced leukemia in all secondary hosts within 13–37 days, further demonstrating efficient serial transplantability and short latency (Fig. S13).

Finally, thymus cells from a leukemic Tg(R:MA9-EGFP) F1 fish with fluorescent thymi were also capable of transmitting disease. Primary transplantation of thymus cells resulted in leukemia in 77% of recipients within 12–47 days, who showed lymphoid or myeloid expansion or abnormal cellular profiles. Secondary transplantation of thymus cells from leukemic primary recipients resulted in leukemia in 56% of recipients within 26–42 days (Fig. S14). Together, these results demonstrate that MA9-driven leukemic cells from both kidney marrow and thymus can be serially transplanted, underscoring the robustness and functional relevance of this zebrafish leukemia model.

We then checked whether the *KMT2A::MLLT3* zebrafish leukemias had acquired additional somatic mutations. We performed whole-exome sequencing (WES) on kidney marrow (KM) cells from six leukemic F0 fish, using matched muscle tissue from the same animals as germline controls. Each exome library yielded approximately 14.4 Gb of sequence, and additional WES data from 20 wild-type zebrafish were used to filter out common population polymorphisms [6]. Applying a somatic *p*-value cutoff of ≤0.001, we identified 395 putative somatic single nucleotide variants and indels across the six leukemias. Among these variants, 16 affected genes with known human homologs (Table S1), including five mutations occurring at positions previously reported as somatically mutated in human cancers. These mutations were found in Stat5, Cyp2j20, Ms4a17a.3, Tapbp.1, and Herc5.3, genes implicated in key cellular processes such as transcriptional regulation, ubiquitination, and cell signaling [7–10]. The presence of these recurring, cancer-associated somatic mutations suggests that they act as cooperating drivers contributing to leukemogenesis and disease progression in our transgenic MA9 zebrafish leukemia model.

We also performed RNA sequencing on flow-sorted viable hematopoietic cells from the whole kidney marrow of seven leukemic and ten wild-type zebrafish. Libraries yielded approximately 10 million reads per sample, amounting to an average of 750 Mb of sequence [11]. Differential expression analysis identified 67 significantly altered transcripts (*q* < 0.05). There were 30 upregulated and 20 downregulated genes among the top 50 differentially expressed transcripts (Fig. S15).

Gene set enrichment analysis revealed significant enrichment of nine signatures, notably involving the KRAS, RAF, MEK, and P53 pathways as well as *MLL* target genes. Genes associated with KRAS/RAF/MEK signaling were upregulated in ZF MA9 leukemias, whereas P53 pathway–associated genes were downregulated in this model [12, 13] (Fig. S16). In addition, a significant enrichment of differentially expressed genes in *MLL* target genes was observed (Fig. S16; Table S2).

Overall, this study presents the first transplantable zebrafish AML model driven by MA9. MA9 expression in early HSCs leads to aggressive leukemia with both myeloid and lymphoid phenotypes, reflecting the heterogeneity seen in patients and murine models [14, 15]. The MA9 ZF model is characterized by early hematopoietic disturbances, has cooperating mutations, and shows transcriptional signatures analogous to human disease, providing a valuable platform for mechanistic studies and preclinical drug testing. Zebrafish allow rapid, medium-throughput drug screening in the whole animal, which is challenging in murine models, highlighting the utility of this system for translational leukemia research.

In conclusion, the Tg(R:MA9) zebrafish model faithfully mimics human *KMT2A::MLLT3* leukemia, offering a versatile tool to dissect conserved leukemogenic pathways, investigate cooperating mutations, and accelerate drug discovery. Its transplantability and germline transmission provide unique opportunities to study leukemia progression, stem cell biology, and potential therapeutic interventions in a cost-effective and efficient vertebrate model.

## Supplementary information

Supplementary Methods and Results

## Data Availability

The datasets generated and/or analysed during the current study are available from the corresponding author on reasonable request. Sequencing data (WES and RNA-seq) have been deposited in NCBI GenBank under accession number SUB16025574.
