# Supplementary material for: A transgenic zebrafish leukemia model driven by KMT2A::MLLT3 (MLL-AF9)
Source: Leukemia. 2026 Apr 22;40(7):1560–3. doi: 10.1038/s41375-026-02953-y (PMC13323063; doi:10.1038/s41375-026-02953-y)
Supplement: Supplementary file 1 — Supplementary Methods and Results [file 41375_2026_2953_MOESM1_ESM.docx]

# **Supplementary Materials and Methods**

## **Construct cloning**

The murine Runx1+23 enhancer/promoter was used to drive expression of the MA9 transgene in hematopoietic stem cells. The Runx1+23 enhancer/promoter was obtained from the Zon lab (Harvard Department of Stem Cell and Regenerative Biology) and the MA9 coding region was derived from pMIR-FLAG-MLL-AF9 (Addgene plasmid #71444). Two Tol2 constructs (pTol2-Runx1+23:MA9-IRES-EGFP-cmlc:EGFP and pTol2-Runx1+23:MA9-IRES-mCherry-cmlc:EGFP) were created using the Gateway cloning system^1^ and the Gateway ® LR Clonase TM Plus Enzyme Mix kit (Invitrogen)(**Fig. S1).** The *MA9* expressing Tol2 constructs were co-injected with Tol2 mRNA into one-cell stage eggs. Twenty four hours after injection, transgene positive larvae were selected based on the GFP expression in their hearts.

## **Construct microinjection**

Transgenic zebrafish were produced by microinjecting pDEST-MLL-AF9 expression constructs (pTol2-Runx1+23:MA9-IRES-EGFP-cmlc:EGFP and pTol2-Runx1+23:MA9-IRES-mCherry-cmlc:EGFP) into one-cell stage wild-type eggs. Larvae were obtained from natural spawnings of AB zebrafish. Microinjections were performed using pre-pulled needles calibrated to deliver 1 nl per pulse. The injection mix contained pDEST plasmid DNA (25 ng/µl), transposase mRNA (25 ng/µl), and Phenol Red as a tracer. At 24 hours post-injection, larvae were screened for GFP expression in the heart. Positive larvae were raised to adulthood under standard husbandry conditions.

**Whole-mount in situ hybridization (WISH)**

RNA whole-mount in situ hybridization was performed on larvae following established protocols. RNA probes were generated by linearizing plasmids containing the relevant cDNA sequences (see Table S1 for probe details). Digoxigenin-labeled antisense probes were synthesized using the SP6/T7 RNA Labeling Kit (Roche). Hybridization signals were visualized using NBT/BCIP or INT/BCIP substrates (Roche). For each probe, two technical replicates were carried out, with approximately 30-40 larvae analyzed per genotype per replicate. Stained larvae were mounted in 100% glycerol and imaged using a Zeiss Axio Zoom microscope. Expression patterns identified by WISH were further validated by reverse transcriptase quantitative PCR (RT-qPCR) analysis.

## **Flow cytometry analysis**

Whole kidney marrow (WKM) of zebrafish was homogenized in ice-cold 0.9 × phosphate-buffered saline (PBS) +5% fetal bovine serum (FBS) and passed through a 40 μm filter. Cells were washed once with the same solution, stained with propidium iodide and analyzed by flow cytometry. Based on the forward scatter (FSC, cell size) and side scatter (SSC, granularity), four distinct populations of erythrocytes, lymphocytes, myeloid, and precursors were identified.

## **Cell transplantation**

Two days before transplantation, recipients were exposed to a sublethal gamma irradiation dose of 18 Gy. On the day of transplantation, whole kidney marrow (WKM) or thymocytes were collected from leukemic fish, followed by counting the viable KM or TC cells using a hemocytometer and Trypan Blue exclusion method. The recipient fish were anesthetized in 4% (v/v) Tricaine, followed by the transplantation of 0.5 million leukemic WKM cells each by intraperitoneal injections.

## **Zebrafish husbandry and ethics**

## All zebrafish (Danio rerio) strains were maintained under standard husbandry conditions and followed the protocols approved by the University of Auckland Animal Ethics Committee (AEC22627). Wild-type (AB) zebrafish were obtained from the Zebrafish International Resource Centre (ZIRC).

## **Stable transgenic lines**

One hundred F0 fish including Tg(Runx1+23:MA9-IRES-EGFP-cmlc:EGFP) (n=53) and Tg(Runx1+23:MA9-IRES-mCherry-cmlc:EGFP) (n=47) were out-crossed with WT fish to identify the F0 fish with germline transmitable MA9 transgene (founder F0 fish) and to establish the transgene positive F1 lines. As a result, six MA9 F1 lines were generated, including five MA9-EGFP lines (a total of n=50 fish) and one MA9-mCherry line (a total of n=30 fish). These transgenic F1 progeny were monitored for leukemia symptoms. From these six F1 lines, two lines, including one MA9-EGFP (a total of n=50 fish) and one MA9-mCherry line (a total of n=22 fish), were maintained as stable lines**.**

## **Histology**

Adult Wild-type and leukemic zebrafish were euthanized on ice and fixed in 4% paraformaldehyde (PFA) at 4 °C for up to 2 weeks. Specimens were decalcified in 0.25% EDTA for two weeks, dehydrated in 75% ethanol, embedded in paraffin, and sectioned at 4 µm thickness. Sagittal sections (4 µm) of paraffin-embedded zebrafish were deparaffinized in 100% xylene (2 × 5 min) and rehydrated through a graded ethanol series (100%, 95%, 70%, 50%) to water. Nuclei were stained with hematoxylin, followed by rinsing in tap water and differentiation in 1% acid alcohol if necessary. Sections were stained in an alkaline solution and counterstained with eosin to label cytoplasm and extracellular matrix. Finally, sections were dehydrated through graded ethanol, cleared in 100% xylene (2 × 5 min), and mounted with a resinous medium before imaging by light microscopy.

## **RT-PCR**

A pool of whole larvae (n=12) or dissected WKM from adult fish was transferred into 300µl Qiagen RLT buffer supplemented with 10% 2-mercaptoethanol (Merck). Total RNA was isolated using Qiagen Rneasy Mini reagents kit (Qiagen GmbH) and RNase-free DNase set (Qiagen GmbH), according to the On-Column DNase Digestion protocol provided by the manufacturer. The RNA was eluted into 20µl RNAse-free water, divided into two fractions( 10µl): one fraction of extracted RNA (10µl) retained as a “no reverse transcriptase” (–RT) control, and the other (10µl) used for first-strand cDNA synthesis reactions using High-Capacity cDNA Reverse Transcription kit (Applied Biosystems). This was followed by PCR amplification using transcript-specific primers, and 2 µl of the (+RT) or (-RT) reverse transcriptase reactions to assess the expression of MA9 at the transcript level (for primers refer to Table S2).

### **RNA extraction and quantitative PCR (qPCR) from zebrafish larvae for WISH validation**

Wild-type and transgenic zebrafish larvae (7–20 larvae, ~30 mg total tissue per sample) were collected at defined developmental stages (24, 48, and 72 hpf). Residual E3 medium was removed, and larvae were lysed in 750 µl TRIzol reagent (Thermo Fisher Scientific) pre-chilled to 4°C. Samples were homogenized using 1 ml and BD insulin syringes, incubated at room temperature (RT) for 5 min, and stored at −70°C or processed immediately.

Total RNA was extracted using a modified TRIzol/RNeasy Mini Kit protocol (Qiagen GmbH). Briefly, 150 µl chloroform was added, samples were vortexed for 15 s, incubated for 3 min at RT, and centrifuged at 13,200 × g for 5 min. The aqueous phase (~400 µl) was mixed with an equal volume of 70% ethanol and loaded onto an RNeasy spin column. After washing with Buffer RW1, on-column DNase digestion was performed using the RNase-Free DNase Set (Qiagen, GmbH) for 15 min at RT. Columns were washed twice with Buffer RPE, and RNA was eluted in 15–20 µl RNase-free water. RNA quality was verified by gel electrophoresis and quantified spectrophotometrically.

cDNA was synthesized from 1 µg total RNA using the High-Capacity cDNA Reverse Transcription Kit (Applied Biosystems). qPCR was carried out using PerfeCTa SYBR Green FastMix (Quantabio) in 10 µl reactions containing 5 µl master mix, 0.5 µl each of 10 µM primers, and 4 µl diluted cDNA. β-actin served as an internal control. Reactions were performed on a QuantStudio Real-Time PCR System (Applied Biosystems) with standard cycling (95°C for 2 min; 40 cycles of 95°C for 15 s and 60°C for 1 min). Relative gene expression was calculated using the ΔΔCt method.

## **Whole exome sequencing**

Whole exome sequencing (WES) was performed using the genomic DNA (gDNA) extracted from KM cells (primary tumor tissue) and muscle cells (germline control) of leukemic fish and from KM cells of WT fish (for internal population specific SNPs). The gDNA samples were sheared using an EpiShear™ Multi-Sample sonicator (Active Motif®, Carlsbad, California, USA) as described by Kakadia et al 6. Two-hundred nanograms of sheared gDNA was used for the preparation of whole exome libraries using the SureSelectXT reagents and SureSelectXT Zebrafish All Exon capture kits following the manufacturer’s protocol (Agilent Technologies, Santa Clara, California, USA). The whole exome libraries were sequenced on a NextSeq® 500 Illumina platform. On average, 40 million paired-end (2 x 150 base pairs) reads were generated per exome. The FASTQ data was aligned to zv9 zebrafish reference genome using bwa, followed by generation of the mpileup files using samtools and parameters specified in Kakadia et. al. ^15^. analysis protocol is provided in supplementary data. VarScan (v2.3.9) was used for tumor-normal comparison (KM vs muscle tissue from the same leukemic fish) to obtain a list of potentially somatic variants in a variant call format (VCF). The following parameters were used for Varscan somatic variant calling: min-coverage 10, min-coverage-normal 10, min-coverage-tumor 8, min-freq-for-hom 0.75, normal-purity 1.0, tumor-purity 0.9, stranf-filter 1 The variants in the output VCF files were annotated using SnpEffect to predict the variants’ effect. Somatic or loss of heterozygosity variants with “High” (e.g. nonsense) or “Moderate” (missense) predicted impact were selected using SnpSift 4.0. Only somatic missense and nonsense variants (SNVs and Indels) with a somatic p-value ≤0.001 were considered for our analysis.

Since histological analysis revealed that the muscle tissue in most of the transgenic leukemic MA9 fish was infiltrated with leukemic cells, we performed WES on DNA pooled from 20 WT zebrafish from our colony to identify common polymorphisms in our fish. We then discarded those putative somatic variants identified by Varscan somatic that were identified as polymorphisms in the WT fish.

## **RNA-Seq analysis**

## Viable hematopoietic cells from the leukemic zebrafish WKM were sorted based on their FSC/SSC characteristics and RNA was isolated using the RNeasy mini kit from Qiagen (Qiagen GmBH). Strand specific cDNA libraries were prepared using the NEXTflex™ Rapid Directional qRNA-seq™ kit (Bioo Scientific) and 10 million 75 bp reads per library were sequenced on an Illumina NextSeq 500 machine. The sequence was analyzed following the Pertea et al. on transcript level expression analysis of RNA-seq experiments with HISAT, StringTie and Ballgown ^3^. A q-value cut-off of <0.05 was used in the Ballgown stattest function.

**Gene set enrichment analysis**

For GSEA, we used GSEA version 4.0.3 to assess the enrichment of oncogenic signatures (189 gene sets), as well as eight leukemia- and AML-specific gene sets, including MLL and MLL-AF9 target gene sets.

**Supplementary Methods tables:**

**Table M.S1: WISH Probes -** Relates to Fig 2A

| **Gene** | **Ref sequence** | **Probe size and gene region** |
| --- | --- | --- |
| *scl* | AF045432.2 | Full transcript (2630 ntds) |
| *gata1a* | NM_131234.2 | Full transcript (1580 ntds) |
| *lyzC* | NM_139180.1 | Full transcript (596 ntds) |
| *mpx* | NM_001351837.1 | Full transcript (3165 ntds) |

**Table M.S2. RT-PCR and qPCR primers relate to Fig. 2, Fig. S2, S6 and S7; qPCR relates to Fig. S7.**

| **Primer name** | **Primer Sequence** | **Expected product size (bp)** |
| --- | --- | --- |
| MF9-junctionR | GCCTTGTCACATTCACCATTC | 238 |
| MF9-junctionF | CGCCCAAGTATCCCTGTAAA |  |
| RT-PCR-EF1a-F | ATCTACAAATGCGGTGGAAT | 188 |
| RT-PCR-EF1a -R | ATACCAGCCTCAAACTCACC |  |
| qPCR-scl -F | CTA TTA ACC GTG GTT TTG CTG G | 103 |
| qPCR-scl -R | CCA TCG TTG ATT TCA ACC TCA T |  |
| qPCR-gata1 -F | AAG ATG GGA CAG GCC ACT AC | 105 |
| qPCR-gata1 -R | TGC TGA CAA TCA GCC TCT TTT |  |
| qPCR-lyz -F | GAAGGACTACGGGATCTTCC | 164 |
| qPCR-lyz -R | CGTTAAGCAAGTCTGAACAGG |  |
| qPCR-mpx-F | TGATGTTTGGTTAGGAGGTG | 123 |
| qPCR-mpx-R | GAGCTGTTTTCTGTTTGGTG |  |
| qPCR-Beta -actin -F | TTC CTT CCT GGG TAT GGA ATC | 104 |
| qPCR-Beta -actin -R | GCA CTG TGT TGG CAT ACA GG |  |

**Table M.S3. Primers used for constructing oncogene vectors**

| **Primer name** | **Primer Sequence** | **Application** |
| --- | --- | --- |
| PMIR-FLAG-MLL-AF9-Fwd-1430 | CACCATGGACTACAAGGACGACG | Generation of a  PCR fragment  encoding the MLL-AF9  ORF. This fragment  was inserted into  pME to generate pME  MLL-AF9 |
| PMIR-FLAG-MLL-AF9-withstop-R | ACCTCGAGTCAGGATGTTCCAGATG |  |

**Supplementary Results:**


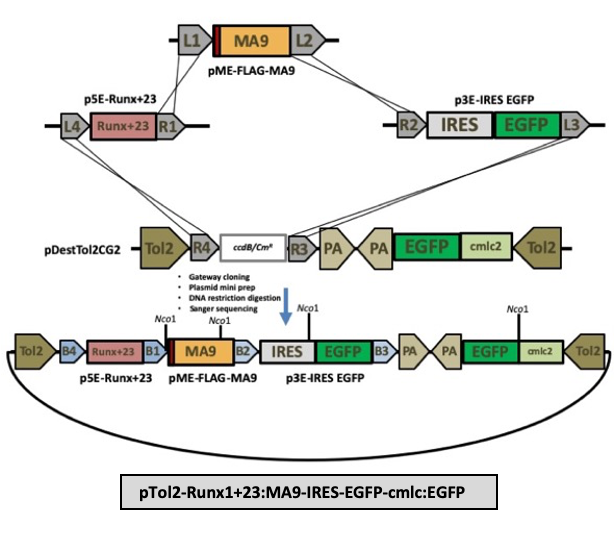


**Fig. S1. Schematic representation of Tol2-Runx1+23:MA9-IRES-(EGFP or mCherry)-cmlc: EGFP construct.** Expression vectors were generated using the three-insert multisite Gateway Tol2Kit system. The murine Runx1+23 enhancer/promoter was cloned into the 5′ entry vector (p5E), the *MLL-AF9 (MA9)* fusion gene into the middle entry vector (pME), and IRES-EGFP or IRES-mCherry into separate 3′ entry vectors (p3E). Recombination of the p5E, pME and p3E vectors with the destination vector pDEST Tol2 CG2 (ID 204; with transgenesis heart marker cmcl:EGFP) was performed according to the manufacturer’s instructions to produce the final pTol2-Runx1+23:MA9-IRES-EGFP-cmlc:EGFP and pTol2-Runx1+23:MA9-IRES-mCherry-cmlc:EGFP constructs. Tol2: Transposase recognition sites used for genomic integration. attB (L/R): Bacterial recombination sites; attB-L indicates the left site, and attB-R indicates the right site. PA: Polyadenylation signal sequence that stabilizes mRNA transcripts. cmlc2: Cardiac myosin light chain 2 promoter, commonly used as a fluorescent reporter marker in the zebrafish heart. IRES: Internal ribosome entry site, allowing bicistronic expression of two open reading frames from the same transcript. FLAG-MA9: FLAG-tagged *MLL-AF9* fusion gene. EGFP: Enhanced green fluorescent protein, used as a reporter. Runx+23: Hematopoietic-specific promoter/enhancer derived from the Runx1+23 regulatory element. ccdB/CmR: Negative selection cassette containing the ccdB gene and a chloramphenicol resistance marker for cloning.


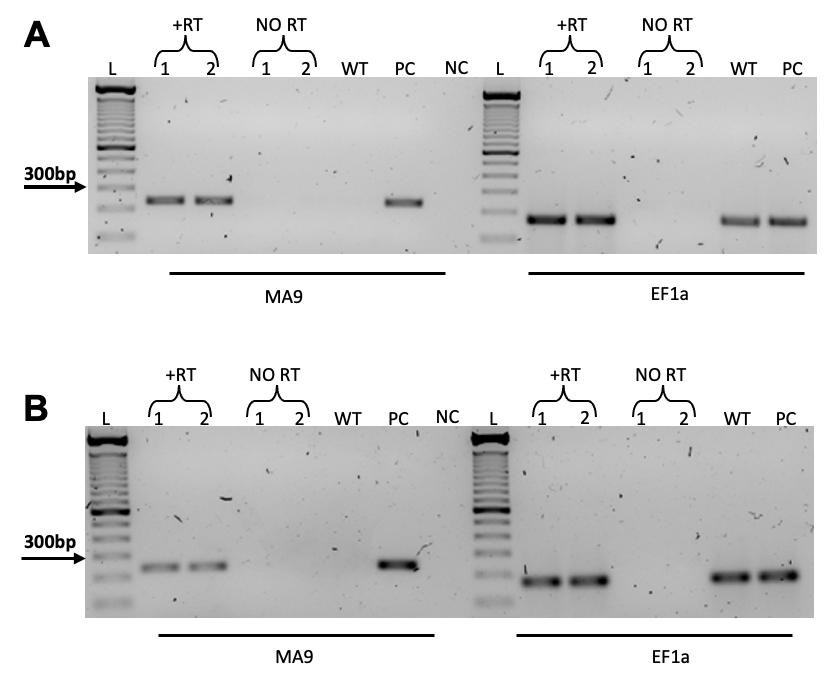


**Fig.S2. RT-PCR confirmation of transgene expression in A) 3 dpf F0 Tg(Runx1+23:MA9) larvae and B) WKM cells from adult F0 Tg(Runx1+23:MA9) animals**. Agarose gels of PCR products generated with gene-specific primers. +RT, reverse transcribed template (cDNA); No RT, RNA template that is Dnase-treated but not reverse transcribed; WT, wild type; PC, PCR positive control for the primers used (with a template of genomic DNA); NC, water. Lanes marked 1, 2 is from RNA obtained from representative F0 animals of the Runx+23:MA9-IRES-EGFP and -mCherry; L, molecular marker (GeneRuler 100 bp ladder,Invitrogen). Expected PCR product sizes: *MA9* (238 bps), *ef1a* product (188 bps). See Table S2 for primer sequences.


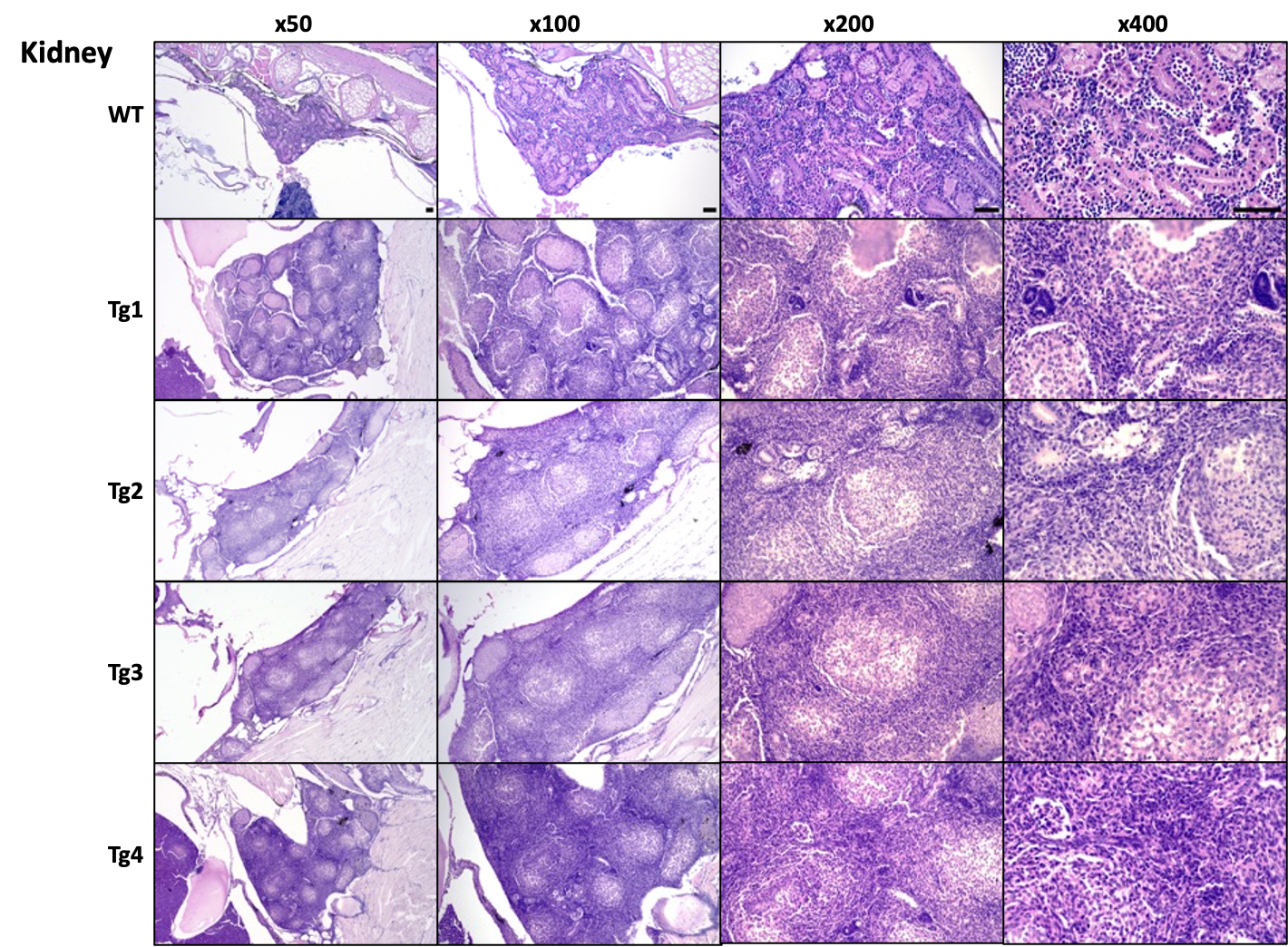


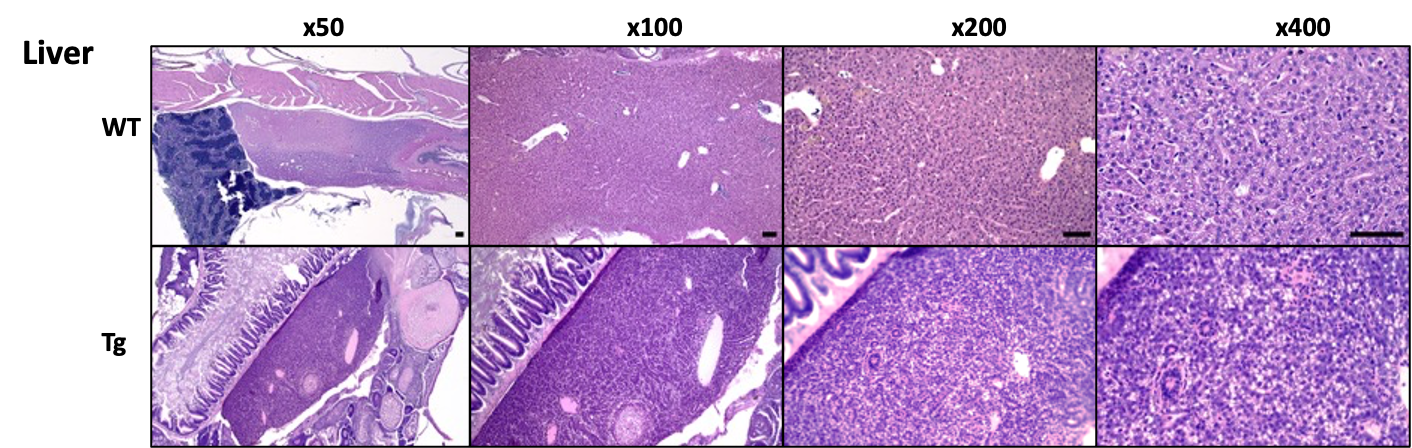


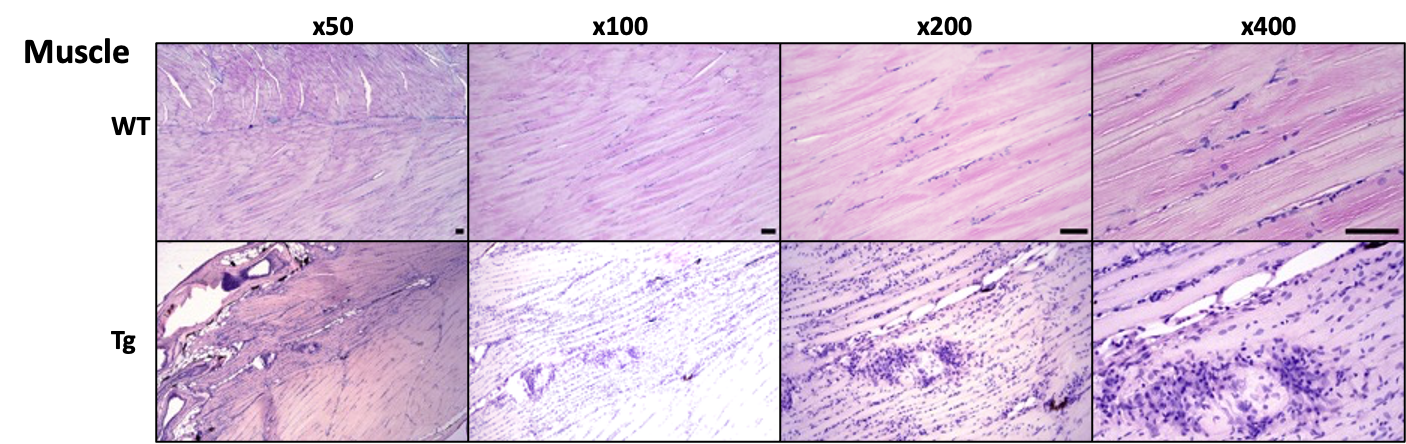


**Fig. S3. Cell infiltration was detected in the kidney, liver, and muscle of F0 Tg(Runx1+23:MA9) leukemic fish**. H&E-stained sections from representative sick R1+23:MA9 F0 animals, illustrating the spectrum of cell densities observed in the kidney, liver, and muscle. Magnification = 50x, 100x, 200x and 400x; Scale bar = 50 μM.

 
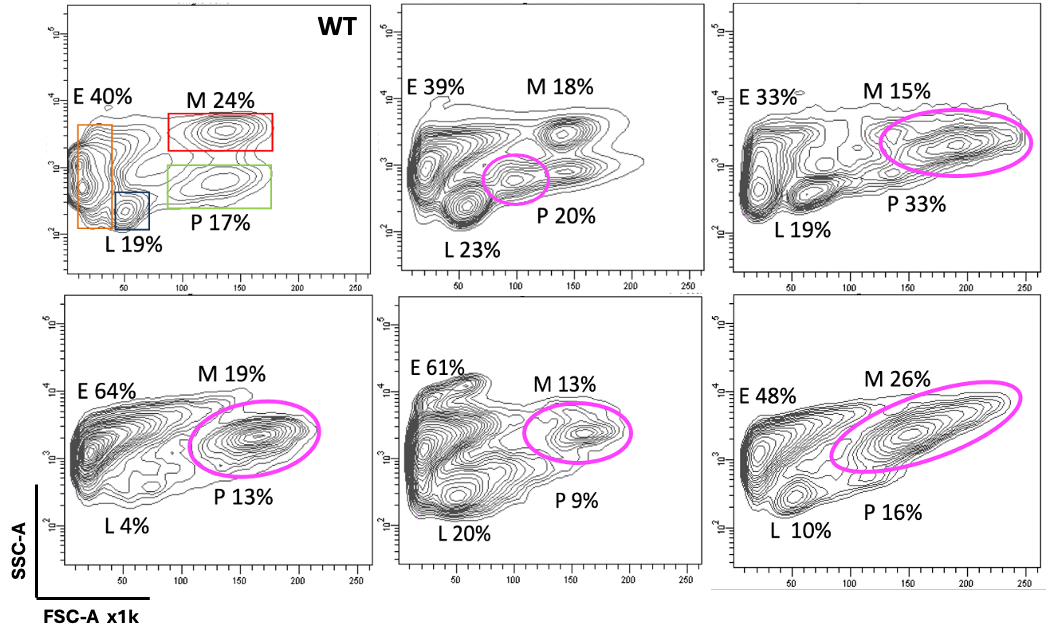


**Fig. S4. WKM flow cytometry profiles of F0 with new subpopulations**

FSC/SSC flow cytometry profiles of individual F0 leukemic animals that had new subpopulations. Cell populations are indicated as M (myeloid), P (precursor), L (lymphoid), and E (erythroid). Newly emerging subpopulations are highlighted with pink circles.


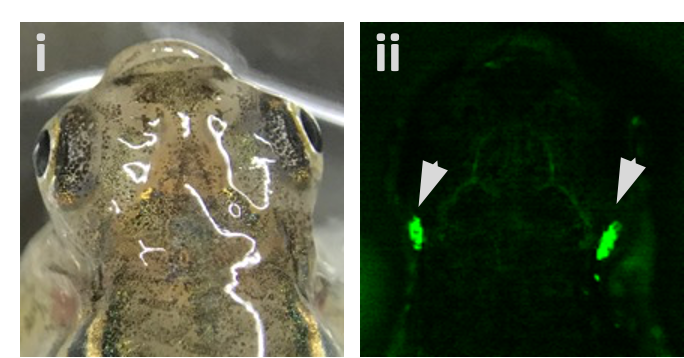


**Fig. S5. GFP expression in the thymus of leukemic Runx1+23:MA9-IRES-EGFP F0 fish**

i) Bright field image of a transgenic F0 Runx1+23:MA9-IRES-EGFP sick fish (11 months) with green thymus. ii) GFP expression in the thymi is seen in the green channel image.


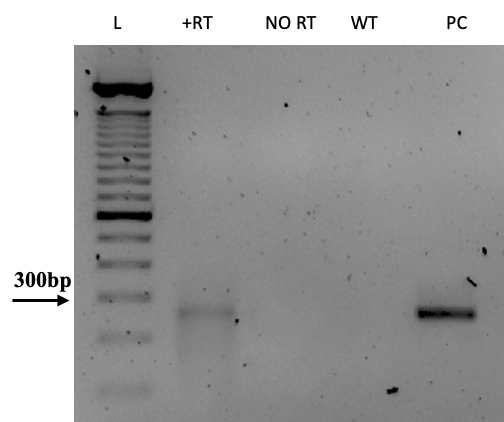


**Fig.S6. MA9 expression was confirmed by RT-PCR in the WKM of adult F1 sick fish**

RNA was obtained from the WKM of representative leukemic F1, and RT-PCR was performed using the MA9 junction-specific primers. Agarose gel representing L: molecular marker (GeneRuler 100 bp ladder,Invitrogen); +RT: reverse transcribed template (cDNA); No RT: RNA template that is Dnase-treated but not reverse transcribed; WT: wild type; PC: PCR positive control for the primers used (with a template of genomic DNA). Expected PCR product sizes: *MA9* (238 bps). See Table S2 for primer sequences.


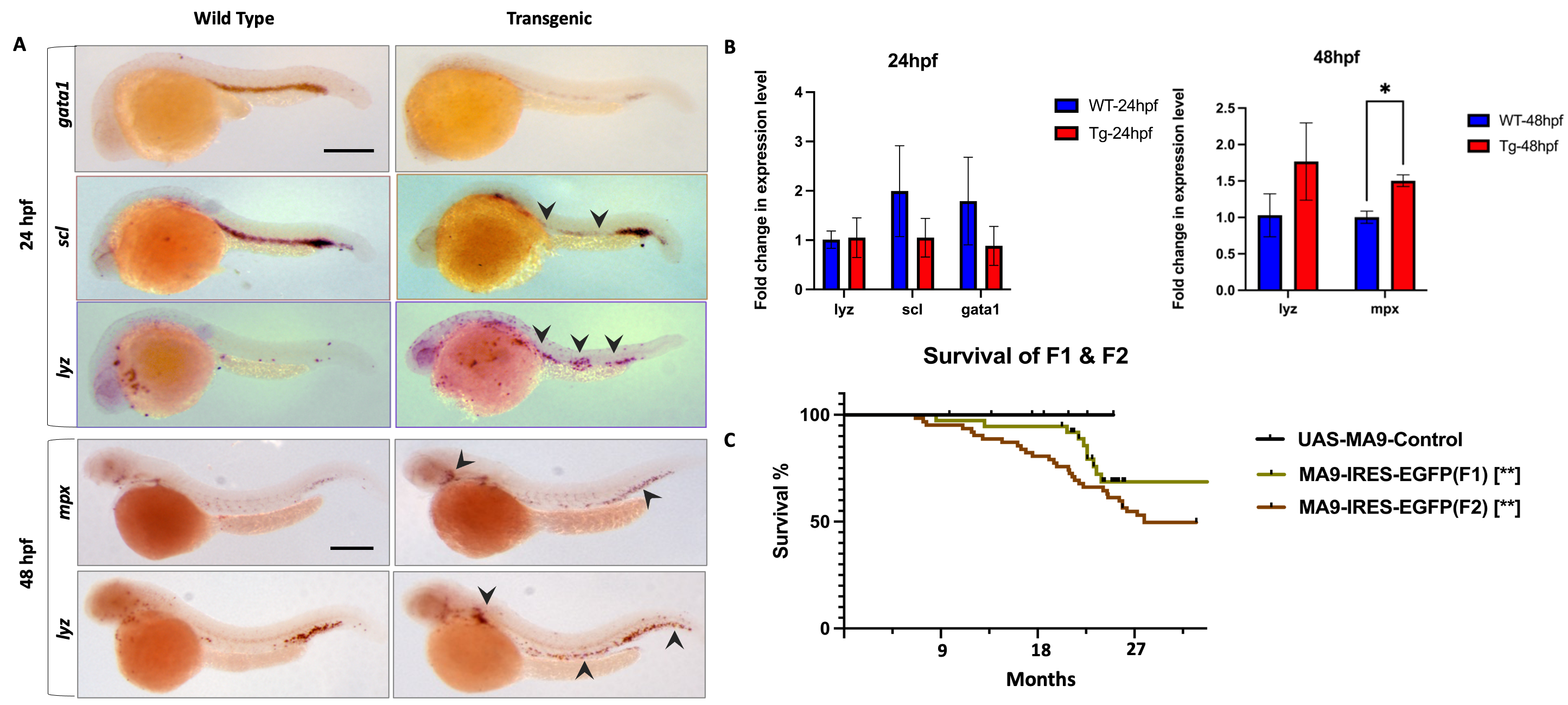


**Fig. S7. MA9 oncogene expression in hematopoietic stem progenitor cells (HSPC) perturbed myeloid fate regulators in early hematopoiesis and caused leukemia in F1 and F2 Tg(Runx1+23:MA9) adult fish**

(A) Whole-mount in situ hybridization (WISH) of F1 larvae at 24 and 48 hours post-fertilization (hpf) using probes for erythroid markers (gata1, scl) and myeloid regulators (mpx, lyz). Transgenic (Tg) larvae displayed reduced expression of erythroid markers at 24 hpf, while expression of myeloid markers was increased at both time points. Black arrows indicate sites of probe signal. Probes used are indicated on the left; genotypes are shown at the top. Scale bar = 200 µm. (B) Quantitative analysis of probe signal intensity, validated by qPCR. Statistical analysis was performed using multiple unpaired t-tests. p = 0.0017. (C) Kaplan–Meier survival analysis of adult F1 and F2 Runx+23:MA9 zebrafish. UAS:MA9-IRES:EGFP fish were used as controls. Tick marks denote censored individuals. Statistical analysis was performed using two-way ANOVA (*p = 0.0021).


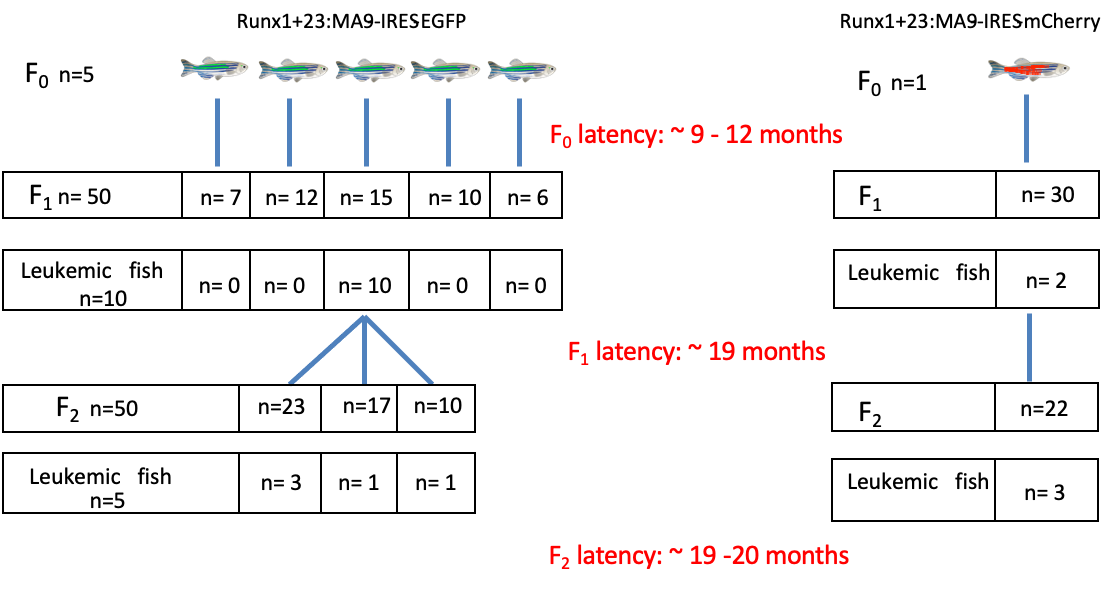


**Fig. S8. Establishment and record of stable F1 and F2 Runx1+23:MA9 transgenic zebrafish lines**

One hundred F0 fish, including Tg(Runx1+23:MA9-IRES-EGFP-cmlc:EGFP) (n=53) and Tg(Runx1+23:MA9-IRES-mCherry-cmlc:EGFP) (n=47), were out-crossed with WT fish to identify the F0 fish with germline transmissible MA9 transgene (founder F0 fish) and to establish the transgene-positive F1 lines. As a result, six MA9 F1 lines were generated, including five MA9-EGFP lines (a total of n=50 fish) and one MA9-mCherry line (a total of n=30 fish). These transgenic F1 progeny were monitored for leukemia symptoms. From these six F1 lines, two lines, including one MA9-EGFP (a total of n=50 fish) and one MA9-mCherry line (a total of n=22 fish), were maintained as stable lines

**
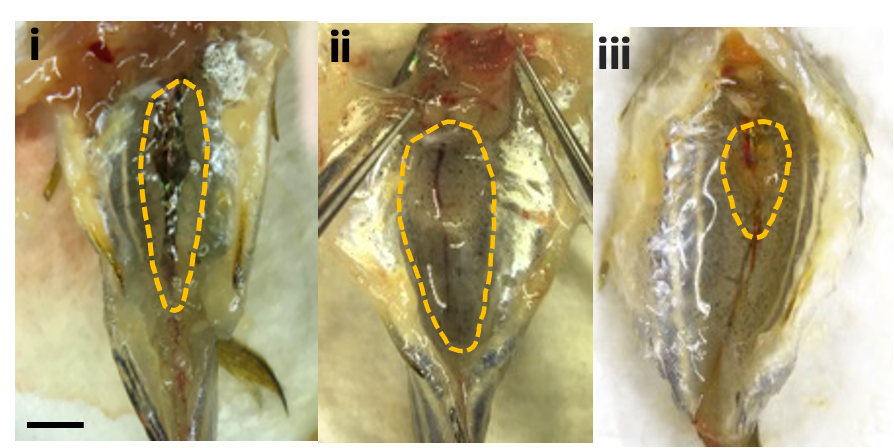
**

**Fig. S9. Dysmorphic kidney in the transplanted MA9 zebrafish.**

MA9 transplanted fish have an expanded pale kidney with a different morphology from the WT kidney. i: WT fish; ii and iii: kidney in Runx+23:MA9 leukemic transplants. The yellow dashed line encircles the kidney. Scale bar is 0.5 cm


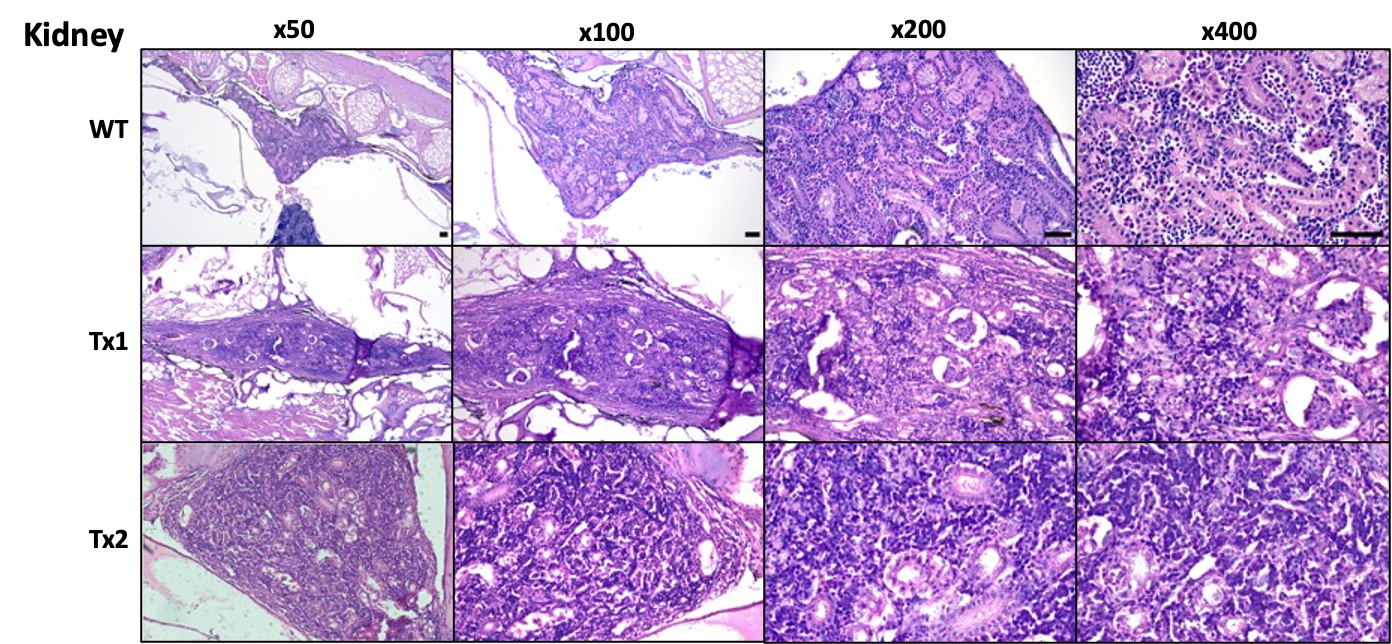

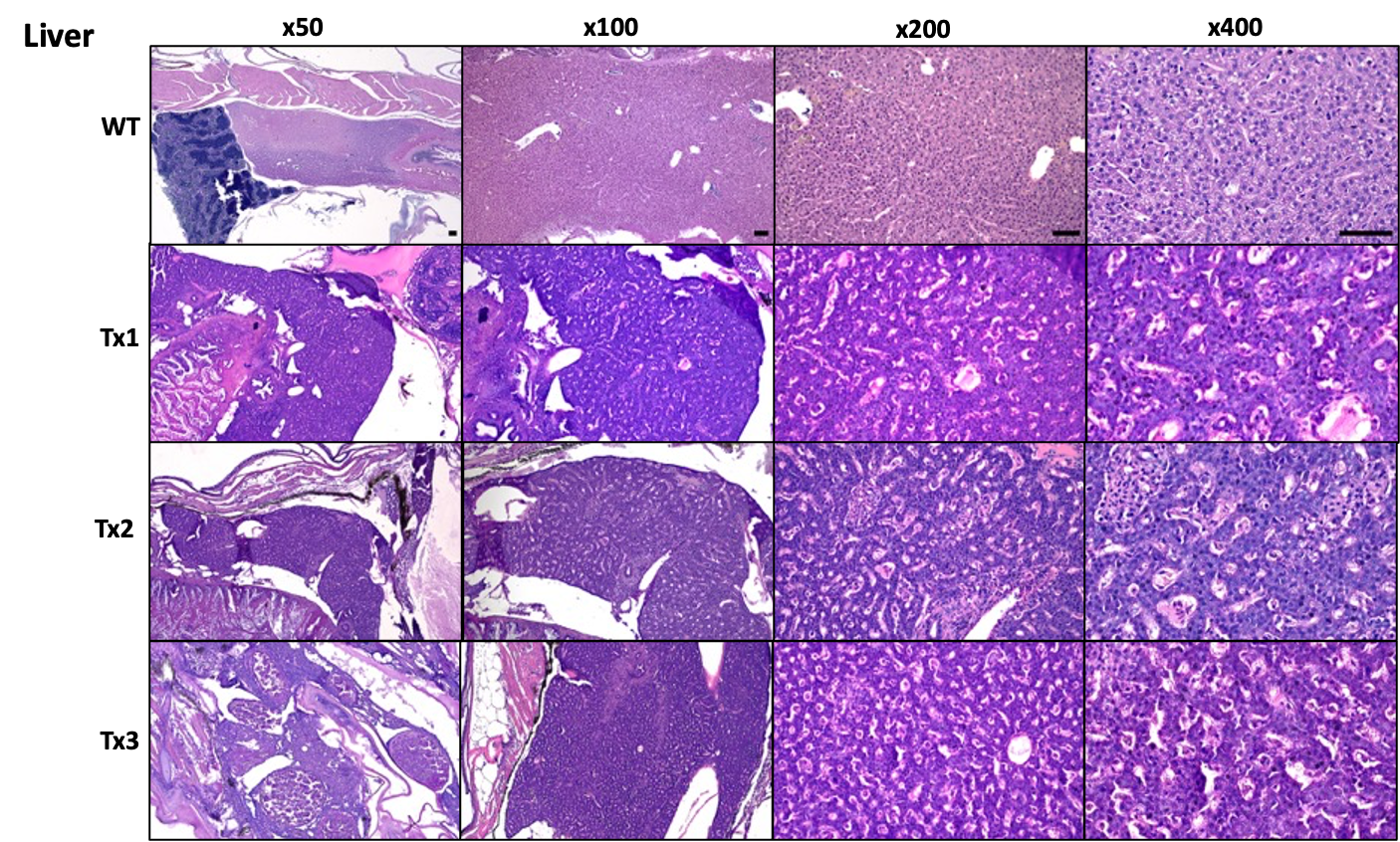


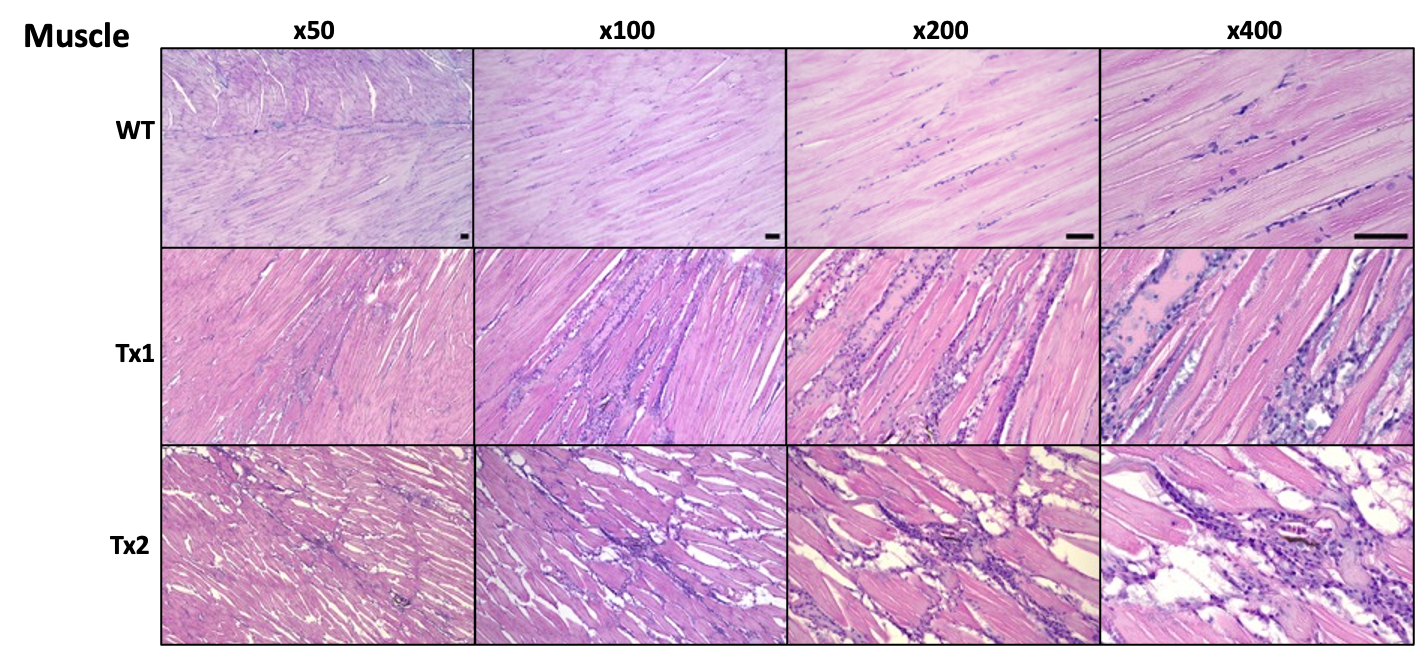


**Fig. S10. Leukemic cellular infiltration in the kidney, liver and muscle of 1° and 2° transplant recipients**

H&E-stained sections from representative sick 1° (Tx1) and 2° (Tx2) transplanted fish, illustrating the spectrum of cell densities observed in the kidney, liver, and muscle. Magnification = 50,100,200 and 400x; Scale bar = 50μM.

**
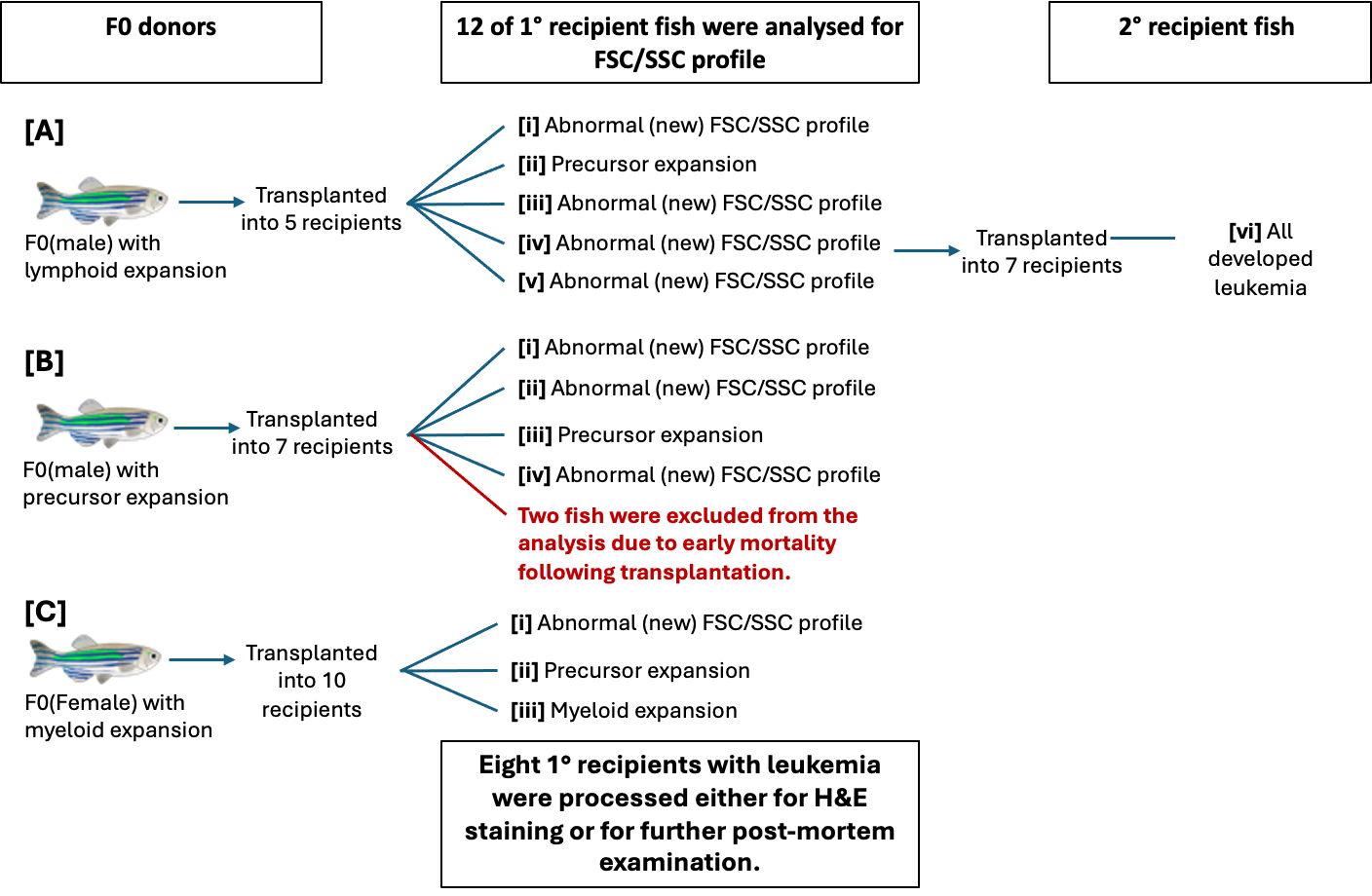
**

**Fig. S11. FSC/SSC profile patterns observed in serially transplanted Runx1+23:MA9 leukemic fish**

FSC/SSC analysis in F0 donors, 12 of 1° recipients, and the 2° recipients showed lymphoid, precursor, myeloid cell expansions or the emergence of an abnormal and new FSC/SSC profile.


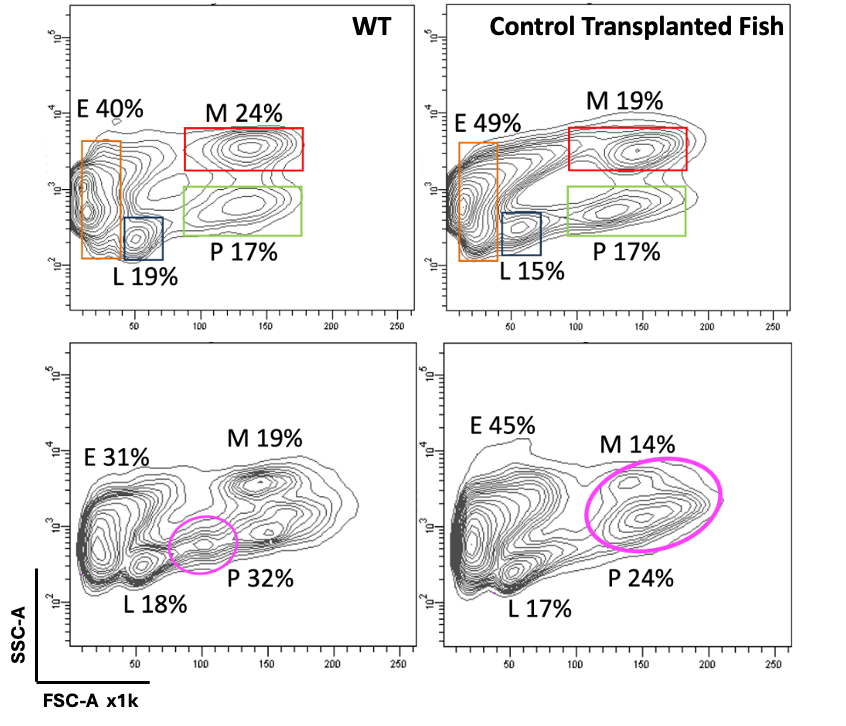


**Fig. S12. WKM flow cytometry of transplanted leukemic fish showed abnormal profiles.**

Representative FSC/SSC flow cytometry plots of secondary (2°) recipient leukemic fish showing abnormal patterns and the emergence of novel cell populations. Cell populations are indicated as M (myeloid), P (precursor), L (lymphoid), and E (erythroid). Newly emerging subpopulations are highlighted with pink circles.


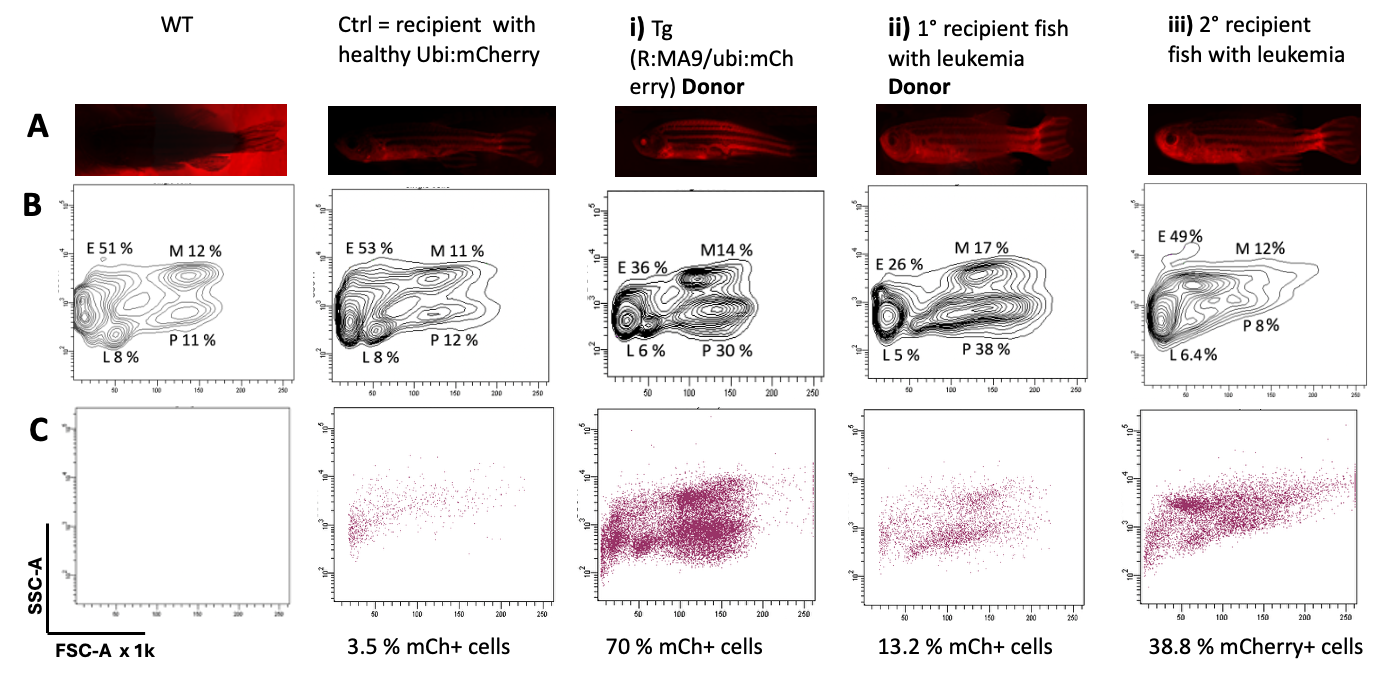


**Fig. S13. WKM flow cytometry profiles of transplanted leukemic fish with mCherry+ cells confirmed leukemia in 1° and 2 recipients**

A) Representative fluorescent image in the red channel shows red mCherry expression in i: Tg(R:MA9/ubi:mCherry), ii: 1° and the iii) 2° recipients; B) FSC/SSC flow cytometry profiles provide a comparison between the WT :wild-type, Ctrl: control recipient transplanted with WKM cells from healthy ubi:mCherry fish, i: Tg(R:MA9/ubi:mCherry), ii: 1° and the iii) 2° leukemic recipients presented in row A. C) FSC/SSC plot of mCherry positive cells of corresponding fish confirmed the transplantation engrftment. The percentage of mCherry+ cells of the individual fish is indicated below the plot.


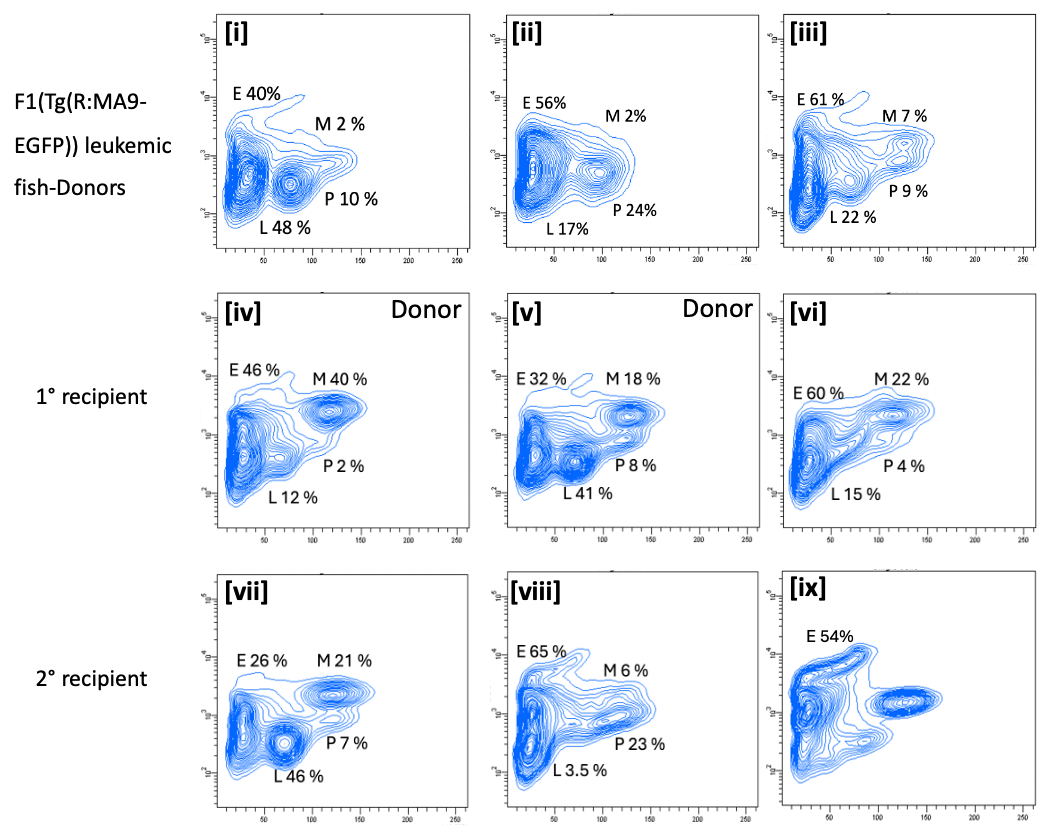


**Fig. S14. WKM flow cytometry profiles of leukemic fish transplanted with thymus cells.**

Representative FSC/SSC flow cytometry profiles of F1 Tg(R:MA9-EGFP) leukemic fish with (i) lymphoid cells expansion, and (ii,iii) abnormal FSC/SSC profiles, used as thymus donors for 1° recipients. Representative FSC/SSC flow cytometry profiles of leukemic 1° recipients with (iv) myeloid cells expansion, (v) lymphoid cells expansion and (vi) abnormal FSC/SSC plots. WKM cells from the iv and v were transplanted into 2° recipients. (vii,viii,ix) representative FSC/SSC profiles of leukemic 2° recipients with (vii) lymphoid cells expansion and (viii) abnormal or, (ix) new cell population emergence. M, P, L, and E correspond to cells in myeloid, precursor, lymphoid, and erythroid gates.


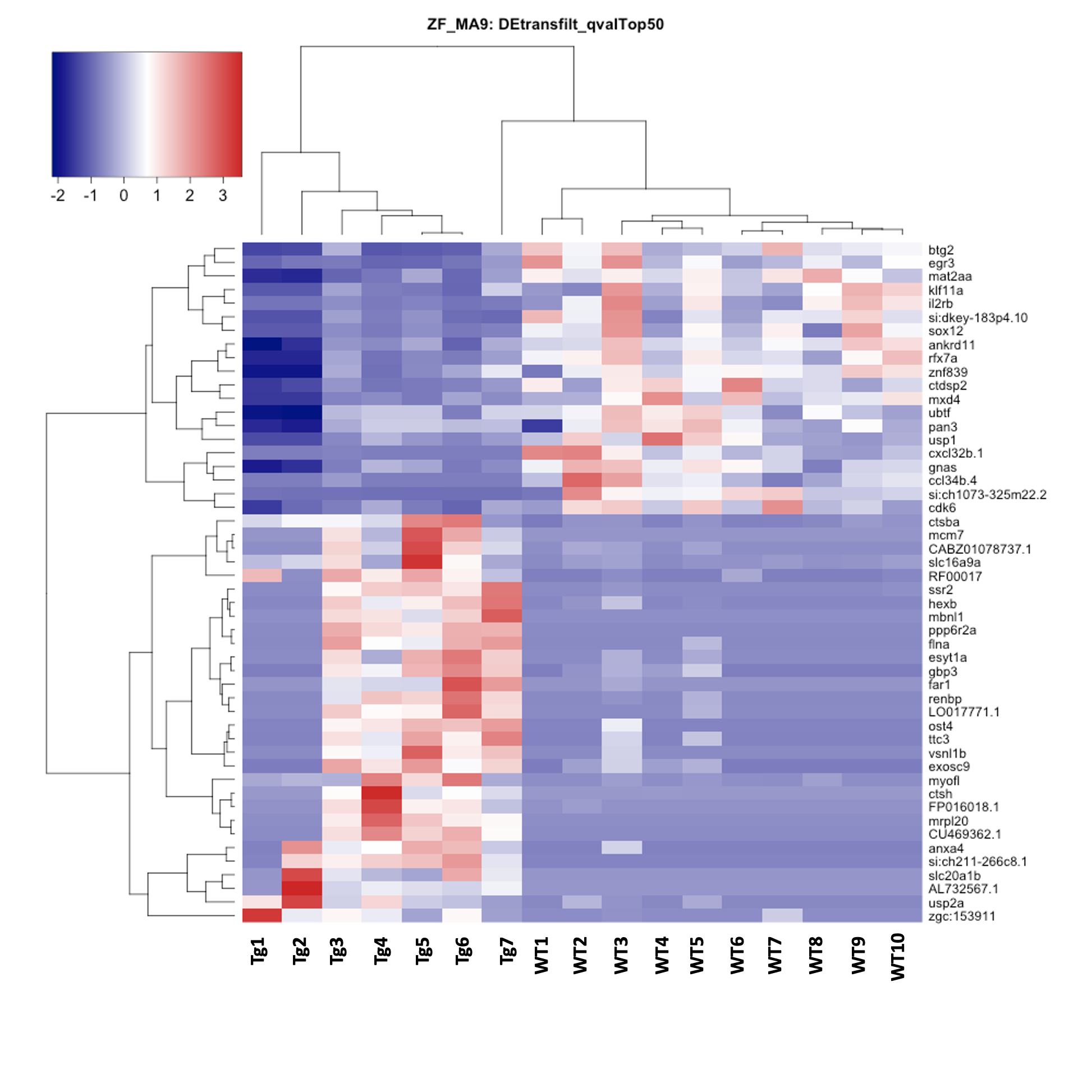


**Fig. S15. Heatmap showing the log 2 fold change of top 50 (q < 0.05) differentially expressed transcripts between leukemic F0 transgenic Tg(Runx1+23:MA9) fish and wildtype (WT) fish.** The heatmap is showing the log2 fold change (log2FC) of twenty downregulated (top rows) and 30 upregulated (bottom rows) transcripts in F0 transgenic Tg(Runx1+23:MA9) fish. Samples (columns) and genes (rows) are hierarchically clustered. Colour scale: blue indicates lower-than-average expression, white indicates average expression, and red indicates higher-than-average expression of a gene in the transgenic fish compared to wildtype fish. Tg = transgenic, WT = Wild type.


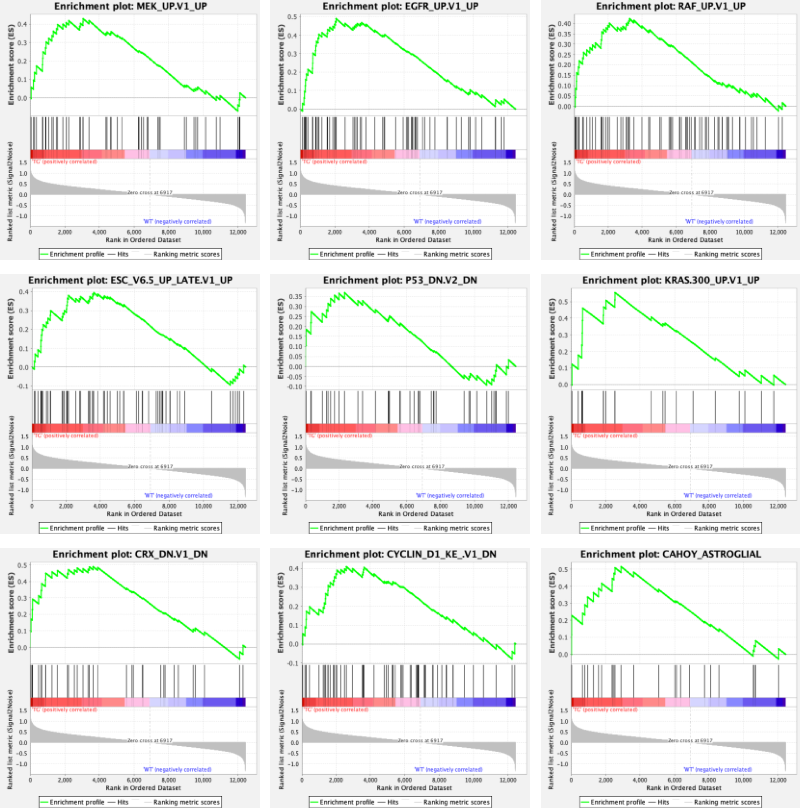


**
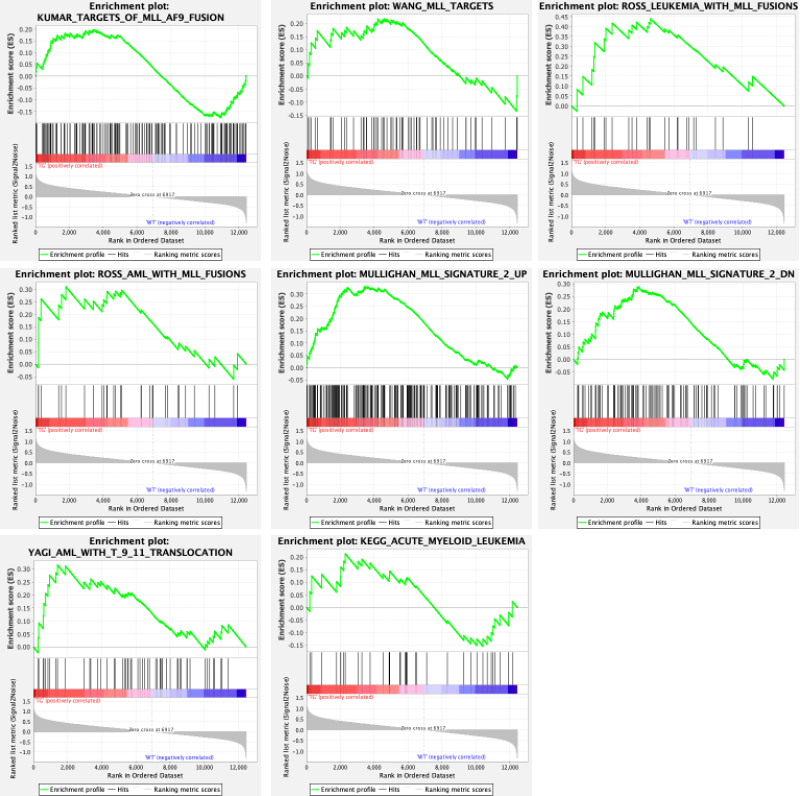
**

**Fig. S16. Gene Set Enrichment Analysis (GSEA) plots showing the enriched pathways in F0**

GSEA analysis using oncogenic signatures (189 gene sets) and leukemia specific genes showed significant enrichment of seventheen gene sets, including the KRAS, RAF, MEK, and P53 gene sets as well as MLL-AF9 target genes, at a nominal P-value of < 0.05.

**Supplementary Result tables:**

**Table S1: Detailed list of 16 final somatic mutations after WES analysis**

| **Gene** | **Genomic location** | **Ref. allele** | **VAR allele** | **AA change** | **Read depth in germline ~ leukemia** | **VAR read germline ~leukemia** | **VAR allele frequency germline ~ leukemia** | **Feature** | **Mutation in Human** | **Reported in** |
| --- | --- | --- | --- | --- | --- | --- | --- | --- | --- | --- |
| *cyp2j20* | chr20:25645221 | C | G | p.Leu462Val | 185~141 | 17~37 | 8.42%~20.79% | ENSDART00000130242.4 | pLeu465Val | lung cancer |
| *stat5.2* | Chr12: 14711202 | A | C | p.Asp396Glu | 13~4 | 0~9 | 0%~69.23% | ENSDART00000124364.5 | p.Glu395Asp p.Glu395* | skin cancer  thyroid cancer |
| *Ms4a17a.3* | chr4:76724408 | T | G | p.Ser2Ala | 296~185 | 57~80 | 16.15%~30.19% | ENSDART00000153867.3 | p.Ser2Phe | skin cancer |
| *tapbp1* | chr19:7039986 | G | A | p.Ser2Ala | 335~ 281 | 3~22 | 15.4%~25.86% | ENSDART00000153867.3 | p.Arg394Cys | large intestine cancer |
| *herc5.3* | chr1:49538270 | C | T | p.Cys50Arg | 592~380 | 128~138 | 17.78%~26.64% | ENSDART00000145960.4 | p.Gly190Arg p.Gly190Glu | lung cancer  skin cancer & lung cancer |
| *ralgds* | chr8: 31810805 | C | A | p.Cys248Phe | 50~28 | 11~32 | 18.03%~53.33% | ENSDARTO0000138959.2 |  |  |
| *pkd1* | Chr1: 54856026 | A | AGTT | p.Thr3460dup | 13~3 | 1~10 | 7.14%~76.92% | ENSDART00000039911.8 |  |  |
| *trim29* | Chr2: 57215029 | A | C | p.His169Pro | 93~76 | 16~61 | 14.68%~44.53% | ENSDART00000137300.3 |  |  |
| *htra2* | Chr10: 35145323 | A | C | p.Asn121His | 49~47 | 4~26 | 7.55%~35.62% | ENSDART00000143954.3 |  |  |
| *ganc* | Chr17: 45427122 | G | C | p.Ala36Pro | 26~4 | 5~11 | 16.13%~73.33% | ENSDART00000109532.5 |  |  |
| *prx* | Chr18: 44627446 | T | C | p.Lys1604Arg | 103~93 | 17~45 | 14.17%~32.61% | ENSDART00000134244.3 |  |  |
| *ppp4cb* | Chr12: 4652161 | C | G | p.Asp82His | 89~42 | 9~19 | 9.18%~31.15% | ENSDART00000149473.2 |  |  |
| *rrp1* | Chr9: 291982 | T | G |  | 27~9 | 1~8 | 3.57%~47.06% | ENSDART00000129585.3 |  |  |
| *klhl2* | Chr1: 19747425 | A | T | p.Tyr9* | 17~6 | 3~13 | 15%~68.42% | ENSDART00000088545.6 |  |  |
| *arhgef4* | Chr2: 16093655 | G | A | p.Pro407Ser | 263~286 | 26~70 | 9%~19.66% | ENSDART00000057216.8 |  |  |
| *dlec1* | Chr4: 20958649 | A | T | p.Asp847Glu | 35~20 | 4~16 | 10.26%~44.44% | ENSDART00000143174.2 |  |  |

Table S2: Enriched gene sets with p-value <0.05 in MA9 zebrafish leukemia deregulated genes

| **Gene set name** | **Description** | **Status in MA9 Zebrafish** | **Reported in** |
| --- | --- | --- | --- |
| EGFR_UP.V1_UP | Genes upregulated in MCF-7 breast cancer cells positive for ESR1 and engineered to express ligand-activatable EGFR | Upregulated | AML M1 to M7 |
| RAF_UP.V1_UP | Genes upregulated in MCF-7 breast cancer cells, overexpressing constitutively active RAF1 gene . | Upregulated | AML & Hairy Cell Leukemia (HCL) |
| MEK_UP.V1_UP | Genes upregulated in MCF-7 breast cancer cells, overexpressing constitutively active MAP2K1 gene | Upregulated | AML & ALL |
| CRX_DN.V1_DN | Genes downregulated in retina cells from Crx knockout mice | downregulated | C/EBPα (member of CRX gene set ) is downregulated in AML |
| CYCLIN_D1_KE_.V1_DN | Genes downregulated in MCF-7 breast cancer cells, over-expressing a mutant K112E form of CCND1 gene . | downregulated | ALL & CLL |
| CAHOY_ASTROGLIAL | Genes upregulated in astroglial cells of developing and mature mouse forebrain | Upregulated |  |
| KRAS.300_UP.V1_UP | Genes upregulated in four lineages of epithelial cell lines over-expressing an oncogenic form of KRAS gene | Upregulated | AML, ALL, CML, MLL rearranged leukemias |
| ESC_V6.5_UP_LATE.V1_UP | Genes upregulated during late stages of differentiation of embryoid bodies from V6.5 embryonic stem cells | Upregulated |  |
| P53_DN.V2_DN | Genes downregulated in HEK293 cells (kidney fibroblasts) upon knockdown of TP53 gene by RNAi | downregulated | AML, ALL, CLL, CML |
| KUMAR_TARGETS_OF_MLL_AF9_FUSION | Genes changed in comparison among the leukemic, preleukemic and wild-type animals, in myeloid leukemia model in mice with germ-line MLL-AF9 fusion knock-in |  | AML |
| WANG_MLL_TARGETS | Genes requiring MLL for H3K4me3 and expression in MEF cells (embryonic fibroblast). |  |  |
| ROSS_LEUKEMIA_WITH_MLL_FUSIONS | Top 100 probe sets associated with MLL fusions irrespective of the lineage of the pediatric acute leukemia. |  |  |
| ROSS_AML_WITH_MLL_FUSIONS | Top 100 probe sets for pediatric acute myeloid leukemia (AML) subtypes with chimeric MLL fusions |  | AML |
| MULLIGHAN_MLL_SIGNATURE_2_UP | Genes up-regulated in pediatric AML (acute myeloid leukemia) with rearranged MLL compared to the AML cases with intact MLL and NPM1 | Upregulated | AML |
| MULLIGHAN_MLL_SIGNATURE_2_DN | Genes down-regulated in pediatric AML (acute myeloid leukemia) with rearranged MLL compared to the AML cases with intact MLL and NPM1 | Downregulated | AML |
| YAGI_AML_WITH_T(9;11)_TRANSLOCATION | Genes specifically expressed in samples from patients with pediatric acute myeloid leukemia (AML) bearing t(9;11) translocation. |  | AML |
| KEGG_ACUTE_MYELOID_LEUKEMIA | Genes aberrantly expressed in acute myeloid patients |  | AML |
